# Cytolethal Distending Toxins Require Components of the ER-Associated Degradation Pathway for Host Cell Entry

**DOI:** 10.1371/journal.ppat.1004295

**Published:** 2014-07-31

**Authors:** Aria Eshraghi, Shandee D. Dixon, Batcha Tamilselvam, Emily Jin-Kyung Kim, Amandeep Gargi, Julia C. Kulik, Robert Damoiseaux, Steven R. Blanke, Kenneth A. Bradley

**Affiliations:** 1 Department of Microbiology, Immunology and Molecular Genetics, University of California, Los Angeles, Los Angeles, California, United States of America; 2 Department of Microbiology, Institute for Genomic Biology, University of Illinois, Urbana, Urbana, Illinois, United States of America; 3 California NanoSystems Institute, University of California, Los Angeles, Los Angeles, California, United States of America; Yale University School of Medicine, United States of America

## Abstract

Intracellular acting protein exotoxins produced by bacteria and plants are important molecular determinants that drive numerous human diseases. A subset of these toxins, the cytolethal distending toxins (CDTs), are encoded by several Gram-negative pathogens and have been proposed to enhance virulence by allowing evasion of the immune system. CDTs are trafficked in a retrograde manner from the cell surface through the Golgi apparatus and into the endoplasmic reticulum (ER) before ultimately reaching the host cell nucleus. However, the mechanism by which CDTs exit the ER is not known. Here we show that three central components of the host ER associated degradation (ERAD) machinery, Derlin-2 (Derl2), the E3 ubiquitin-protein ligase Hrd1, and the AAA ATPase p97, are required for intoxication by some CDTs. Complementation of Derl2-deficient cells with Derl2:Derl1 chimeras identified two previously uncharacterized functional domains in Derl2, the N-terminal 88 amino acids and the second ER-luminal loop, as required for intoxication by the CDT encoded by *Haemophilus ducreyi* (Hd-CDT). In contrast, two motifs required for Derlin-dependent retrotranslocation of ERAD substrates, a conserved WR motif and an SHP box that mediates interaction with the AAA ATPase p97, were found to be dispensable for Hd-CDT intoxication. Interestingly, this previously undescribed mechanism is shared with the plant toxin ricin. These data reveal a requirement for multiple components of the ERAD pathway for CDT intoxication and provide insight into a Derl2-dependent pathway exploited by retrograde trafficking toxins.

## Author Summary

Cytolethal distending toxins (CDTs) are produced by several bacterial pathogens and increase the ability of these bacteria to cause disease. After being taken up by host cells, CDTs are trafficked to the endoplasmic reticulum (ER) where they must translocate across the ER membrane to gain access to their intracellular target; however, this translocation process is poorly understood for CDTs. Here we provide evidence that CDTs require components of the ER-associated degradation (ERAD) pathway, a normal cellular process utilized to translocate terminally misfolded ER lumenal and membrane proteins across the ER membrane for degradation in the cytosol. Deletion of a key member of this pathway, Derl2, makes cells resistant to multiple CDTs. Interestingly, two domains within Derl2 which are required for ERAD of misfolded proteins are dispensable for intoxication by CDT. Further, we report two previously uncharacterized domains within Derl2 that are each required for intoxication. Consistent with a role of Derl2, abrogation of two other members of the ERAD pathway, Hrd1 and p97, results in retention of CDT in the ER and resistance to intoxication. Taken together, these data provide novel insight into how CDTs exit the ER and therefore gain access to their cellular targets.

## Introduction

Cytolethal distending toxins (CDTs) are produced by a variety of Gram-negative pathogens including the oral pathogen *Aggregatibacter actinomycetemcomitans*, the sexually transmitted pathogen *Haemophilus ducreyi*, and the gastrointestinal pathogens, *Escherichia coli* and *Campylobacter jejuni*. These toxins belong to a larger, emerging group of intracellular-acting “cyclomodulins” whose expression is associated with increased persistence, invasiveness and severity of disease [1]–[7]. Rather than inducing overt cytotoxicity and tissue damage, cyclomodulins drive more subtle alterations in the host through changes in cell cycle progression. CDTs cause DNA damage in susceptible host cells, resulting in the induction of DNA repair signaling mechanisms including phosphorylation of the histone H2AX, cell cycle arrest at the G_2_/M interface and disruption of cytokinesis [8]. Inhibiting the cell cycle interferes with many functions of rapidly dividing eukaryotic cells, including lymphocytes and epithelial cells, which play a role in immunity and provide a physical barrier to microbial pathogens [5],[9],[10]. In cultured cells, the DNA damage response ultimately leads to apoptotic cell death, while *in vivo*, persistent DNA damage may give rise to infection-associated oncogenesis [11]. Although the cellular response to CDTs is well characterized [8], [12], the mechanism by which CDTs bind to host cells and ultimately gain access to their nuclear target is less clear.

CDTs generally function as complexes of three protein subunits, encoded by three contiguous genes (*cdtA*, *cdtB*, *cdtC*) in a single operon [13]. Consistent with the AB model of intracellular acting toxins [14], CdtB functions as the enzymatic A-subunit and possesses DNase I-like activity responsible for inducing DNA damage within the nuclei of intoxicated cells [15], [16]. CdtA and CdtC are thought to function together as the cell-binding B-moiety of AB toxins to deliver CdtB into cells [17]–[20].

To exert their cyclomodulatory effects, CDTs must be taken up from the cell surface and transported intracellularly in a manner that ultimately results in localization to the nucleus. Recent data suggest that the endosomal trafficking pathways utilized by CDTs from unrelated pathogens are different, but that all CDTs are trafficked in a retrograde manner through the Golgi apparatus and into the ER [21], [22]. CDTs and other retrograde trafficking toxins lack the ability to translocate themselves across the ER membrane and must therefore rely on host cellular processes to access their intracellular targets. Toxins such as cholera toxin, Shiga toxin, and ricin use a host-encoded protein quality control process known as ERAD [23]–[31]. ERAD is a normal physiological process by which misfolded proteins in the ER lumen and membrane are translocated to the cytoplasm for degradation by the proteasome. The core machinery driving ERAD in mammalian cells consists of the Hrd1/Sel1L ubiquitin ligase complex, the Derlin family of proteins and may also involve Sec61 [32]. Translocation of misfolded proteins across the ER membrane is energetically unfavorable and is facilitated by the AAA-ATPase p97 [33]–[35]. While toxins use various components of the ERAD pathway to exit the ER lumen, they avoid proteasomal degradation, thereby hijacking the host quality control mechanism to gain access to the cytosol.

In contrast to other retrograde trafficking toxins, several reports have suggested that ERAD does not play a role in the translocation of CDT across the ER membrane. Mutant cell lines deficient in the retrotranslocation of several retrograde trafficking toxins, such as cholera toxin, *Pseudomonas aeruginosa* exotoxin A, *E. coli* heat labile-toxin IIb, plasmid encoded toxin, and ricin were sensitive to CDT [22], [36]. Overexpression of Derlin-GFP fusions, which can act as dominant negative proteins to inhibit ERAD, did not block CDT intoxication [22]. Thermal stability of CdtB suggested that this catalytic subunit does not unfold prior to translocation and thus may not be an ERAD substrate [37]. Finally, CdtB was not found in the cytoplasm of intoxicated cells prior to nuclear localization, but rather was localized with ER membrane projections into the nucleus (i.e. nucleoplasmic reticulum), leading to the model that CDTs translocate directly from the ER lumen into the nucleoplasm [37]. Contrary to these data, others have described requirements for nuclear localization signals within the CdtB subunits, implicating a requirement for retrotranslocation to the cytosol prior to trafficking to the nucleus [38]–[40]. Identifying host factors required for translocation of CDT across the ER membrane would provide insight into mechanism of toxin entry; however, these data have been elusive [22], [41], [42].

Here we describe the results of two genetic screens aimed at identifying host genes required for intoxication by CDT from four human pathogens. These results implicate key components of the ERAD pathway in retrotranslocation of CDT and thereby provide insight into the mechanism by which host cells are intoxicated by this family of bacterial toxins.

## Results

### Derl2 is required for intoxication by CDT

**Figure 1:**
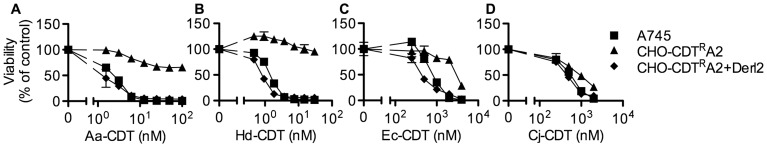
The chemically mutagenized clone, CHO-CDT^R^A2, is resistant to CDT and complemented by expression of Derl2. Parental A745 cells, chemically induced mutant CHO-CDT^R^A2 cells, and CHO-CDT^R^A2 cells expressing Derl2 were seeded in a 384-well plate (1×10^3^ cells/well) and allowed to adhere overnight, followed by 48 hour intoxication with Aa-CDT (a), Hd-CDT (b), Ec-CDT (c) and Cj-CDT (d) and quantitation of viability using ATPlite 1-step reagent (Perkin Elmer). Data are representative of at least three independent experiments performed in triplicate, percent viability is normalized to unintoxicated controls and error bars indicate standard error.

In order to identify genes that confer sensitivity to CDT, we performed two separate forward somatic cell genetic screens. First, we utilized the frameshift mutagen ICR-191 to induce mutations in ten separate pools of CHO-pgs A745 cells (A745). Each pool of 1×10^6^ cells was selected with 20 nM *A. actinomycetemcomitans* CDT (Aa-CDT), a toxin concentration high enough to cause death in parental cells. Five of the ten pools yielded Aa-CDT resistant clones; the most resistant clone isolated (CHO-CDT^R^A2) was resistant to the highest dose of Aa-CDT tested (Fig. 1a). Interestingly, CHO-CDT^R^A2 cells were also resistant to the highest dose of *H. ducreyi* CDT (Hd-CDT) tested (Fig. 1b) and more modestly resistant to CDTs from *E. coli* (Ec-CDT; Fig. 1c) and *C. jejuni* (Cj-CDT; Fig. 1d). To identify the gene responsible for CDT resistance in CHO-CDT^R^A2 cells, we utilized a high throughput cDNA expression-based complementation approach. A custom cDNA library consisting of approximately 3.7×10^3^ arrayed clones was prepared from the mammalian gene collection [43]. Plasmid DNA was isolated from the library, normalized for concentration, plated individually into 384-well plates and reverse transfected into CHO-CDT^R^A2 cells. After 72 hours, the transfected cells were intoxicated with 20 nM Aa-CDT and immunostained using fluorescent anti-pH2AX antibodies to identify activation of CDT-mediated DNA damage response. Cells were stained with Hoechst 33342 to enumerate nuclei, imaged by automated fluorescence microscopy and scored using automated image analysis software. We identified *Mus musculus Derlin-2* (Genbank ID: BC005682), a gene involved in the ERAD pathway, as able to complement the sensitivity of CHO-CDT^R^A2 cells to Aa-CDT. CHO-CDT^R^A2 cells were transduced with a retroviral vector encoding Derl2 to verify this finding and test whether Derl2 was able to complement resistance to the remaining three CDTs. CHO-CDT^R^A2 cells expressing Derl2 regained sensitivity to all four CDTs tested to near parental levels (Fig. 1a–d).

**Figure 2:**
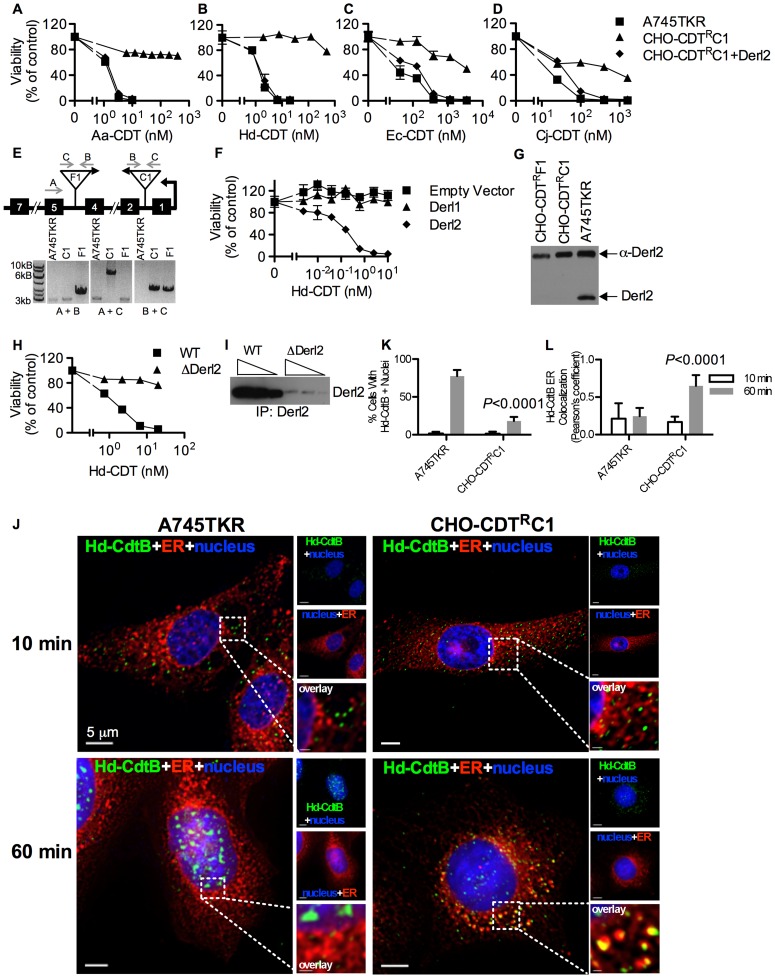
Derl2 is required for CDT intoxication. Viability of parental A745TKR cells, retrovirally induced mutant CHO-CDT^R^C1 cells, and CHO-CDT^R^C1 cells expressing Derl2 after intoxication with Aa-CDT (a), Hd-CDT (b), Ec-CDT (c) and Cj-CDT (d). Intoxication was performed similar to Fig. 1. (e) Top: representation of the *Derl2* open reading frame with boxes representing exons, gray arrows representing primers, and upside down triangles representing proviral insertions. Bottom: agarose gel of genomic PCR from parental A745TKR, CHO-CDT^R^C1 and CHO-CDT^R^F1 cells using primers detailed in the diagram. (f) Overexpression of Derl1 does not complement resistance to CDT. Derl2 deficient CHO-CDT^R^C1 cells expressing empty vector, Derl1, and Derl2 were intoxicated with Hd-CDT, similar to Fig. 1. (g) Derl2 was immunoprecipitated from normalized cell lysates and precipitated proteins analyzed by western blot with anti-Derl2 antibody. (h) CRISPR mediated deletion of Derl2 in HeLa cells causes resistance to Hd-CDT. HeLa cells were transfected with Cas9 DNA and gDNA, followed by selection with G418 and Hd-CDT. Following selection, wildtype and Derl2-deleted cells were intoxicated with Hd-CDT, similar to figure 1. (i) CRISPR mediated deletion of Derl2 results in decreased expression as judged by western blot of anti-Derl2 immunoprecipitated protein from normalized cell lysates. Increasing amounts of immunoprecipitated protein loaded for each condition, corresponding to input from 0.5, 1, or 2×10^6^ cells. (j–l) Retrograde trafficking of Hd-CDT in Derl2 deficient cells is blocked at the endoplasmic reticulum. (j) A745TKR and CHO-CDT^R^C1 cells were incubated with Hd-CDT on ice, washed and incubated at 37°C for 10 or 60 minutes. Cells were then fixed and stained with DAPI (nuclei, blue), Concanavalin A (ER, red) and α-Hd-CdtB (green) antibody. White scale bars indicate 5 µm. (k,l) Quantification of microscopy results comparing the percentage of cells with at least one green puncta localized to the nucleus or Pearson's coefficient values indicating colocalization of the Hd-CdtB signal with the ER marker. Images and quantitation are representative of those collected from a total of 30 randomly chosen cells analyzed during three independent experiments and error bars represent standard deviations.

In a parallel effort to identify genes required for CDT intoxication, a retroviral mutagenesis approach was employed [44]. Approximately 1×10^7^ A745 cells expressing the tetracycline repressor protein fused to the Krüppel associated box from human *Kox1* (A745TKR) were transduced with murine leukemia virus (MLV) encoding the tetracycline repressor element at a multiplicity of infection of 0.1 and selected with 5 nM Hd-CDT, a toxin concentration high enough to cause death in parental cells. Two independent pools produced Hd-CDT-resistant clones. Subsequent characterization of one clone from each pool, CHO-CDT^R^C1 and CHO-CDT^R^F1, revealed that they were resistant to cell killing by the highest concentrations of the four CDTs tested (Fig. 2a–2d) as well as cell cycle arrest induced by lower CDT concentrations (Fig. S1). The site of mutational proviral integration was determined using a combination of sequence capture, inverse PCR and sequencing [44]. Proviral integration sites in the mutants were distinct; the mutagenic integration in CHO-CDT^R^C1 cells occurred between the first and second *Derl2* exons and occurred in the opposite orientation in CHO-CDT^R^F1 cells between the fourth and fifth *Derl2* exons (Fig. 2e). Overexpression of Derl2 in these mutants complemented sensitivity to all CDTs tested (Fig. 2a–2d, S2). In contrast, overexpression of the functionally related Derl1, which shares 51% homology and 35% amino acid identity with Derl2, failed to complement sensitivity to Hd-CDT in CHO-CDT^R^C1 cells (Fig. 2f). Both CHO-CDT^R^C1 and CHO-CDT^R^F1 mutant cells displayed decreased Derl2 expression by immunoprecipitation followed by western blot (Fig. 2g). Targeted deletion of Derl2 was performed in HeLa cells using the Cas9 clustered regularly interspaced short palindromic repeats (CRISPR) system [45]. HeLa cells lacking Derl2 were resistant to Hd-CDT (Fig. 2h, 2i). Additionally, siRNA mediated knockdown of Derl2 in HeLa cells rendered them resistant to Hd-CDT (data not shown). Although the demonstration of a direct physical interaction between Derl2 and CDT would support the hypothesis that Derl2 is part of a retrotranslocation apparatus, attempts to co-immunoprecipitate CDT with Derl2 were unsuccessful, likely due to very small quantities of CDT reaching the ER during intoxication.

Although Derlins have been most intensely studied as important factors in the translocation of ERAD substrates, these proteins have also been implicated in the trafficking of the plant toxin ricin from endosomes to the Golgi apparatus [46]. To identify which step of the CDT retrograde trafficking pathway was blocked in Derl2-deficient cells, the intracellular trafficking of Hd-CDT in parental A745TKR and mutant CHO-CDT^R^C1 and CHO-CDT^R^F1 cells was assessed by immunofluorescence microscopy as a function of time. After 10 minutes of intoxication, Hd-CdtB was clearly internalized into all the cell types tested (Fig. 2j–2l, S3). However, after 60 minutes, significantly more CdtB had localized to the nucleus of the parental A745TKR cells than in the Derl2-deficient CHO-CDT^R^C1 and CHO-CDT^R^F1 cells. In the CHO-CDT^R^C1 and CHO-CDT^R^F1 cells, Hd-CdtB was clearly localized to the ER, even after 60 minutes, but nearly absent within the ER of the parental A745TKR cells. Together, these data support a model that Derl2 is required for retrograde translocation of Hd-CdtB from the ER lumen.

### Hrd1 is required for intoxication by CDT

**Figure 3:**
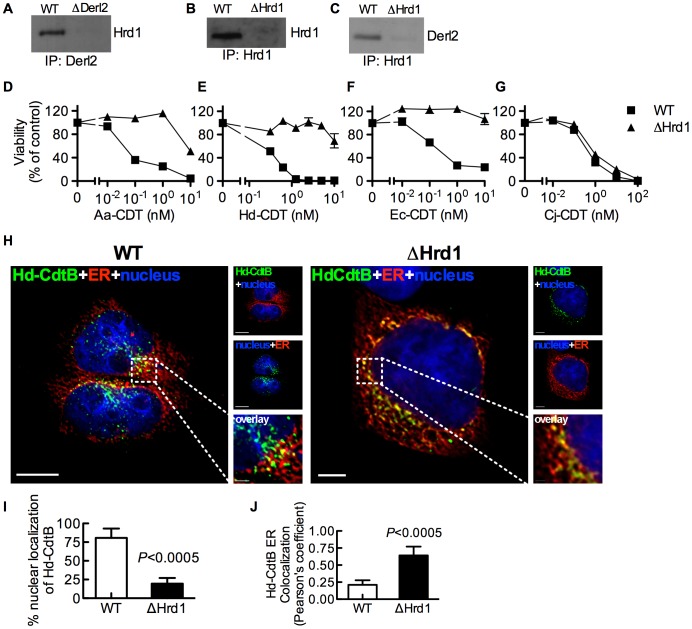
Hrd1 is required for CDT intoxication. (a) Co-immunoprecipitation of Derl2 and Hrd1. Derl2 was immunoprecipitated as in figure 2i and samples were analyzed for Hrd1 by western blot. (b) CRISPR mediated deletion of Hrd1 (ΔHrd1) results in decreased expression as judged by western blot of Hrd1 from α-Hrd1 immunoprecipitated protein from normalized cell lysates. (c) Co-immunoprecipitation of Derl2 with Hrd1. Hrd1 was immunoprecipitated and samples were analyzed for Derl2 by western blot. (d–g) Wild type 293 and ΔHrd1 cells were intoxicated with Aa-CDT (d), Hd-CDT (e), Ec-CDT (f) and Cj-CDT (g) similar to figure 1. Percent viability is normalized to unintoxicated controls and error bars indicate standard error. (h–j) Retrograde trafficking of Hd-CDT in ΔHrd1 cells is blocked at the endoplasmic reticulum. pDsRed2-ER (red) transfected 293 cells and ΔHrd1 cells were incubated with Hd-CDT on ice, washed and incubated at 37°C for 240 minutes. Cells were then fixed and stained with DAPI (nuclei, blue) and α-Hd-CdtB (green) antibody. White scale bars indicate 5 µm. (i,j) Quantification of microscopy results comparing the percentage of cells with at least one green puncta localized to the nucleus (i), or Pearson's coefficient values indicating colocalization of the Hd-CdtB signal with the ER (j). Images and quantitation are representative of those collected from a total of 30 randomly chosen cells analyzed during two independent experiments and error bars represent standard deviations. Unless otherwise noted, data are representative of at least three independent experiments.

Derl2 is part of the Hrd1-containing “retrotranslocon”, a protein complex that mediates retrotranslocation of ERAD substrates [47]. Indeed, Hrd1 was co-immunoprecipitated with Derl2 from wildtype but not Derl2-deficient cells (Fig. 3a). Similarly, Derl2 could be co-immunoprecipitated from wildtype cells, but not from cells in which Hrd1 was targeted by CRISPR (Fig. 3b–3c). Intoxication of Hrd1-deficient cells revealed that this gene, like Derl2, is required for cell killing by multiple CDTs (Fig. 3d–3g, S4). Interestingly, cells lacking Hrd1 displayed full sensitivity to intoxication by Cj-CDT (Fig. 3g). Similar to Derl2 deficient cells, deletion of Hrd1 resulted in retention of Hd-CDT in the ER 240 minutes post-intoxication (Fig. 3h–3j). These data suggest that the Derl2 and Hrd1-containing retrotranslocon is required for intoxication by multiple CDTs, implicating a role for the ERAD pathway in cellular entry for a subset of this family of toxins.

### Retrotranslocation of CdtB is distinct from previously characterized ERAD Substrates

**Figure 4:**
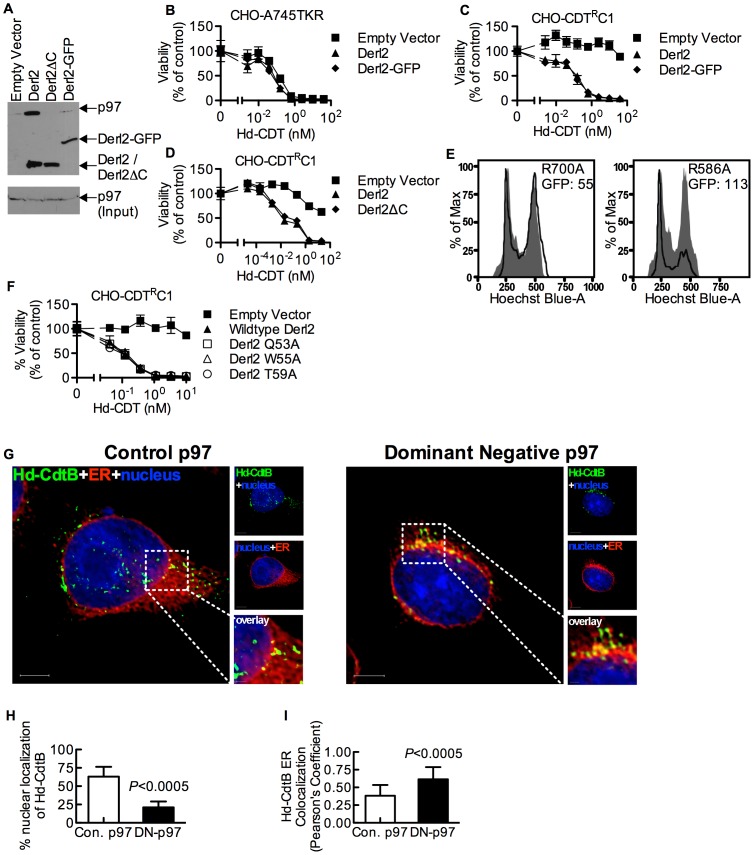
The interaction of Derl2 and p97 is not required for CDT intoxication. (a) Derl2-GFP fails to bind p97, similar to Derl2ΔC. 293 cells were transfected with vectors encoding S-tagged versions of the indicated forms of Derl2. After 3 days, the cells were lysed and western blot was performed on S-protein precipitates with anti-p97 and anti-S-tag antibodies (b) Overexpression of Derl2-GFP does not affect Hd-CDT intoxication of parental A745TKR cells. Parental A745TKR cells expressing empty vector, Derl2 or Derl2-GFP were intoxicated with Hd-CDT, similar to Fig. 1. (c, d) Derl2-GFP and Derl2ΔC complement sensitivity to Hd-CDT in CHO-CDT^R^C1. CHO-CDT^R^C1 cells expressing empty vector, Derl2, (c) Derl2-GFP or (d) Derl2ΔC were intoxicated similar to Fig. 1. (e) Dominant negative p97 reduces sensitivity of 293 cells to Hd-CDT. 293 cells stably expressing TCRαGFP were transfected with plasmids encoding CD4 and either dominant negative (R586A) or control (R700A) p97, followed by intoxication with Hd-CDT for 48 hours and staining with Hoechst and anti-CD4 antibodies. Flow cytometry was performed to obtain geometric mean fluorescence values for TCRαGFP (GFP) in CD4+ cells and cell cycle profile of CD4 negative (grey shaded; untransfected control) and CD4 positive cells (black lines). (f) The Derl2 WR motif is not required for intoxication by Hd-CDT. CHO-CDT^R^C1 cells expressing empty vector, wildtype Derl2, Derl2 Q53A, Derl2 W55A or Derl2 T59A were intoxicated similar to figure 1. (g–i) Retrograde trafficking of Hd-CDT in p97 deficient cells is blocked at the endoplasmic reticulum. (g) Following transfection with pH2B-GFP (blue) and either dominant negative or control p97, wildtype and ΔHrd1 cells were incubated with Hd-CDT on ice, washed and incubated at 37°C for 240 minutes. Cells were then fixed and stained with anti-Hd-CdtB (green) antibody and anti-calreticulin antibody (red). White scale bars indicate 5 µm. pH2B-GFP pseudo-colored blue; Hd-CdtB pseudo-colored green and calreticulin pseudo-colored red (h, i) Quantification of microscopy results comparing the percentage of cells with at least one green puncta localized to the nucleus or Pearson's coefficient values indicating colocalization of the Hd-CdtB signal with the ER. Images and quantitation are representative of those collected from a total of 30 randomly chosen cells analyzed during two independent experiments and error bars represent standard deviations. Unless otherwise noted, data are representative of at least three independent experiments, percent viability is normalized to unintoxicated controls and error bars indicate standard error.

Derlins have been implicated in retrotranslocation of misfolded proteins out of the ER [35], [48]. In order to evaluate whether Derl2 might function by a similar mechanism to retrotranslocate CDTs, we investigated the importance of several Derlin functional motifs required for the retrotranslocation of previously characterized ERAD substrates. A carboxyl terminal SHP box (FxGxGQRn, where n is a non-polar residue) was recently demonstrated to be required for the interaction of Derlins with the AAA ATPase p97 [49], which provides energy to extract ERAD substrates from the lumen into the cytosol [33], [34], [50]. To assess the importance of p97-Derl2 interactions for the escape of CdtB from the cytosol, we tested whether Derl2 with a deletion of the C-terminus (Derl2ΔC) that removes the SHP box could complement Derl2 deficiency in CHO-CDT^R^C1 cells. Additionally, we tested a dominant negative form of Derl2 with a C-terminal GFP tag (Derl2-GFP)[22], [48]. Similar to what had been shown previously, Derl2ΔC was unable to bind p97 (Fig. 4a) [47], [49]. Further, Derl2-GFP was also unable to bind p97 (Fig. 4a). Surprisingly, intoxication studies revealed that despite failing to interact with p97, Derl2-GFP did not act as a dominant negative inhibitor, and that both Derl2-GFP and Derl2ΔC complemented sensitivity to Hd-CDT (Fig. 4b–d). These results suggest that Hd-CDT has evolved to use a Derl2-dependent retrotranslocation pathway that is independent of interaction between Derl2 and p97.

Although the interaction between Derl2 and p97 is not required for Hd-CDT retrotranslocation, this does not preclude a requirement for p97 in intoxication. To investigate this, dominant negative (R586A) and control (R700A) versions of p97 were overexpressed in 293 cells. Activity of the dominant negative p97 was confirmed by an increase in fluorescence signal from the ERAD substrate TCRαGFP [51] (Fig. 4e). Expression of dominant negative p97 caused a reduction in cell cycle arrest in G2 mediated by Hd-CDT, compared to control p97 (Fig. 4e). Consistent with a role for p97 in egress of CdtB from the ER lumen, expression of the dominant negative p97 resulted in retention of Hd-CDT in the ER after 240 minutes of intoxication (Fig. 4g–4i).

We next evaluated the importance of a second functional domain required for Derl2-mediated retrotranslocation of ERAD substrates. Derlins were recently classified as members of the rhomboid protease family of proteins, although they lack key residues required for proteolytic activity [49]. Rhomboid proteases are unique in that they contain an aqueous membrane-embedded cavity that allows for hydrolytic catalysis within the lipid bilayer [52]. Similar to other rhomboid proteases, Derl2 contains a “WR motif” (Q/ExWRxxS/T) in the sequence between the first and second transmembrane domains and a GxxxG motif in the sixth transmembrane domain. The WR motif protrudes laterally into the bilayer and plays a role in rearrangement of the local lipid environment [52], [53] while GxxxG motifs enable intra- and inter-molecular dimerization of transmembrane domains [52], [53]. Mutation of either of these domains in Derl1 renders it unable to retrotranslocate a constitutively misfolded protein to the cytosol for proteosomal degradation [49]. To test for a role for these motifs in CDT egress from the ER, Derl2 variants with single point mutations in the residues that comprise the WR and GxxxG motifs were expressed in Derl2 deficient CHO-CDT^R^C1 cells. Expression of Derl2 variants Q53A, W55A and T59A complemented the resistance to Hd-CDT in CHO-CDT^R^C1 cells to the same levels as that of wildtype Derl2 (Fig. 4f). One point mutant in the WR domain (R56A) and mutants in either residue of the GxxxG domain (G175V, G179V) failed to complement CHO-CDT^R^C1 cells; however, these mutants were poorly expressed as determined by immunoprecipitation and western blot, and therefore no conclusion can be made regarding a role for these residues (data not shown). These data suggest that although the WR motif is required for retrotranslocation of misfolded proteins by Derl1 [49], it is not required for retrotranslocation of Hd-CDT.

### Identification of Derl2 domains that support intoxication by Hd-CDT

**Figure 5:**
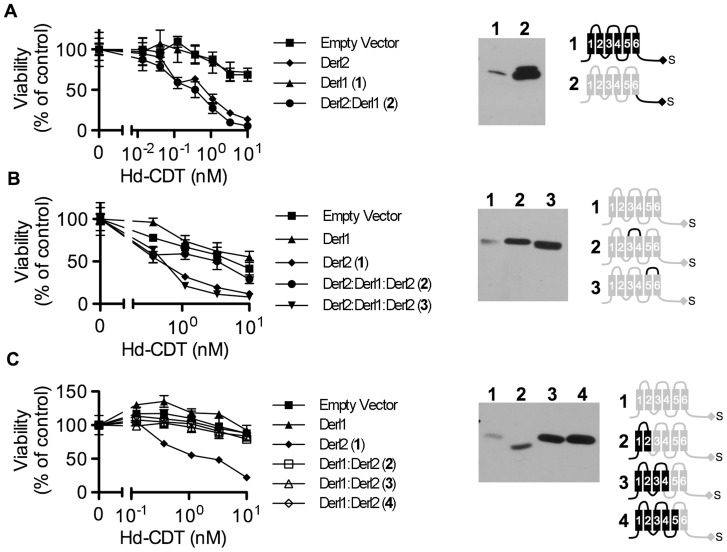
Identification of Derl2 domains required for CDT intoxication. (a–c) CHO-CDT^R^C1 cells expressing empty vector (squares), Derl1-S (triangles), or Derl2-S (diamonds) were intoxicated in each panel, similar to Fig. 1, and compared to derlin variants indicated below. Anti-DERL1 (a) or anti-Derl2 (b, c) western blot of S-protein agarose precipitated protein from normalized cell lysates show expression levels of chimeric derlins. Cartoons depict Derl1 (black) and Derl2 (grey) sequences in each chimera. (a) CHO-CDT^R^C1 cells expressing Derl1-S (triangles, #1) or Derl2^1–187^:Derl1^189–251^-S tag (circles, #2) were challenged with Hd-CDT. (b) CHO-CDT^R^C1 cells expressing Derl2-S (diamonds, #1), Derl2^1–112^:Derl1^114–121^:Derl2^120–239^-S (circles, #2) or Derl2^1–161^:Derl1^163–171^: Derl2^171–239^-S (inverted triangles, #3) were intoxicated as above. (c) CHO-CDT^R^C1 cells expressing Derl2-S (diamonds, #1), Derl1^1–88^:Derl2^88–239^-S (open boxes, 2), Derl1^1–138^:Derl2^138–239^-S (open triangles, #3) or Derl1^1–162^:Derl2^162–239^-S (open diamonds, #4) were intoxicated as above. Data are representative of at least three independent experiments performed in triplicate, percent viability is normalized to unintoxicated controls and error bars indicate standard error.

In order to provide insight into the mechanism by which Derl2 supports intoxication, we set out to identify Derl2 domains that are required for intoxication by Hd-CDT. Taking advantage of the knowledge that Derl1 is sufficiently divergent from Derl2 such that it cannot complement Derl2 deficiency (Fig. 2f), we constructed chimeric proteins comprised of fusions between homologous segments of Derl1 and Derl2 to map Derl2 segments that support intoxication by Hd-CDT. Replacing the C-terminal cytoplasmic tail of Derl2 with that from Derl1 (Derl2^1–187^:Derl1^189–251^) gave a chimera that retained function and complemented sensitivity to Hd-CDT in CHO-CDT^R^C1 cells, consistent with a dispensable role for this domain (Fig. 5a). Likewise, CHO-CDT^R^C1 cells expressing a fusion protein in which the third ER luminal loop of Derl2 was replaced with that from Derl1 (Derl2^1–112^:Derl1^114–121^:Derl1^120–239^) were sensitive to Hd-CDT, indicating that this domain is not required for intoxication (Fig. 5b).

In contrast, two distinct domains were identified in Derl2 that were each independently required for intoxication by Hd-CDT. Three fusion proteins comprised of Derl1 from the N-terminus through the second, fourth and fifth transmembrane domains respectively fused to the remaining portions of Derl2 (Derl1^1–88^:Derl2^88–239^; Derl1^1–138^:Derl2^138–239^; Derl1^1–162^:Derl2^162–239^) were unable to complement sensitivity to Hd-CDT in CHO-CDT^R^C1 cells, implicating a Derl2-specific sequence within the first 88 N-terminal residues as required for CDT intoxication (Fig. 5c). Second, a fusion protein consisting of Derl2 with the second ER luminal loop of Derl1 (Derl2^1–161^:Derl1^163–171^: Derl2^171–239^) was unable to complement sensitivity, demonstrating that one or more of the six amino acids in the second luminal loop unique to Derl2 were also required for intoxication by Hd-CDT (Fig. 5b). We attempted to express several other Derl1:Derl2 chimeric proteins; however, these were expressed at levels lower than their wildtype counterparts and therefore these results were deemed inconclusive (data not shown). Taken together, these data identify two distinct domains of Derl2 required for Hd-CDT intoxication.

### Derl2 and Hrd1 contribute to but are not required for sensitivity to ricin

**Figure 6:**
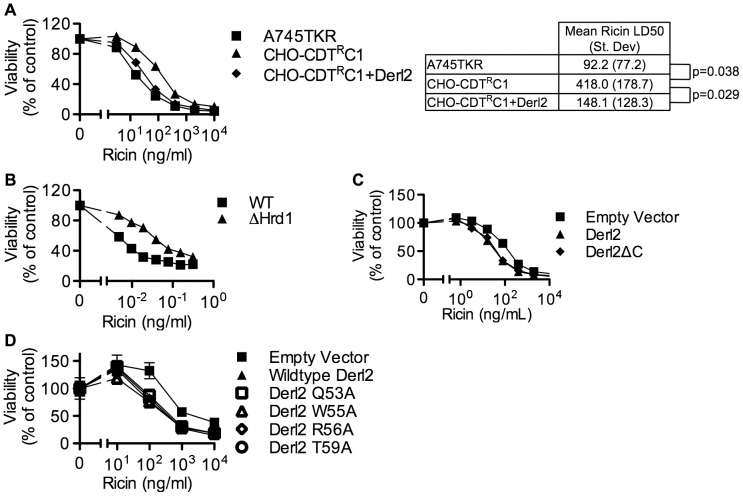
Derl2 and Hrd1 contribute to sensitivity to Ricin, independent of the Derl2 WR motif and the interaction of Derl2 with p97. (a) Derl2 deficiency causes resistance to ricin. A745TKR cells, CHO-CDT^R^C1 cells, and CHO-CDT^R^C1 cells expressing Derl2 were seeded in a 384-well plate (1×10^3^ cells/well) and allowed to adhere overnight, followed by 48 hour intoxication with ricin and quantitation of viability using ATPlite 1-step reagent (Perkin Elmer). Ricin LD_50_ values were calculated from three independent experiments and paired t-test was performed to calculate two tailed p-values. (b) CRISPR mediated Hrd1 deletion in 293 cells causes resistance to ricin. Wildtype and Hrd1-deleted 293 cells were intoxicated with ricin, similar to figure (a). (c) Derl2ΔC complements the resistance to ricin. CHO-CDT^R^C1 cells expressing empty vector, Derl2 and Derl2ΔC were intoxicated similar to (a). (d) The Derl2 WR motif is not required for intoxication by ricin. CHO-CDT^R^C1 cells expressing empty vector, wildtype Derl2, Derl2 Q53A, Derl2 W55A and Derl2 T59A were intoxicated similar to (a). Data are representative of at least three independent experiments performed in triplicate, percent viability is normalized to unintoxicated controls and error bars indicate standard error.

Similar to CDT, several other protein toxins such as ricin, Shiga toxin and cholera toxin rely on retrograde trafficking from the cell surface through the ER in order to gain access to the cytoplasm [25], [54]. Recently, RNAi-mediated repression of members of the Derlin family was shown to cause a slight resistance to ricin [26], [46] that was attributed to reduced trafficking from endosomes to the Golgi apparatus [46]. Similarly, the Derl2 deficient mutant cell line CHO-CDT^R^C1 displayed four-fold resistance to ricin, which was complemented by transduction with Derl2 (Fig. 6a). CRISPR mediated deletion of Hrd1 in 293 cells caused resistance to ricin, albeit to a lesser degree than resistance to Hd-CDT (Fig. 6b, 2h). This low-level resistance to ricin suggests that Derl2 and Hrd1 contribute to, but are not absolute requirements for ricin intoxication. In contrast, a high level of resistance to multiple CDTs resulted from Derl2 or Hrd1 deficiency (Fig. 2). Interestingly, the novel Derl2 SHP box- and WR motif-independence characterized for CDT was shared with ricin. Derl2ΔC and Derl2 WR mutants were able to restore sensitivity to Derl2 deficient CHO-CDT^R^C1 cells (Fig. 6c, 6d), suggesting that Derl2 may have multiple functions that are independent of the conserved WR motif and SHP box-mediated interactions with p97.

## Discussion

In order to gain access to their intracellular targets, retrograde trafficking toxins such as CDT bind the plasma membrane, are endocytosed and then trafficked though endosomes, the Golgi apparatus and ultimately the ER. At this point they must cross the formidable barrier posed by the host cellular membrane. The current model is that retrograde trafficking toxins commandeer the host ERAD pathway to cross the ER membrane, thereby gaining access to the cytosol. Various components of the ERAD machinery have been identified for cytoplasmic delivery of ricin, Shiga, and cholera toxins as well as for *Pseudomonas aeruginosa* exotoxin A [23]–[29]. These ERAD components include members of the HRD ubiquitin ligase complex, Hrd1 and Sel1L [27], [28], Derlins 1–3 [26], [30], [31], ER proteins involved in substrate recognition and unfolding of ERAD substrates [23]–[25], and the Sec61 translocon [26], [29]. Interestingly, different toxins appear to require distinct ERAD components, suggesting that multiple pathways exist by which toxins are translocated out of the ER lumen [26]. In contrast to these toxins, the pathway(s) by which CDTs exit the ER and ultimately gain access to the host nucleus was previously unknown. An ERAD-independent pathway was suggested based on failure of Derl1-GFP and Derl2-GFP fusion proteins to block intoxication by Hd-CDT, as well as susceptibility of mutant cells to CDT that were resistant to multiple other retrograde trafficking toxins [22], [36]. Here we provide evidence that three core components of the ERAD machinery, Derl2, Hrd1 and p97, are in fact required for intoxication by multiple CDTs and that abrogation of these key members of the ERAD pathway leads to Hd-CDT accumulation in the ER, consistent with a role in retrotranslocation.

The inability of Derl1 to complement Derl2 deficiency further enabled identification of novel domains within Derl2 required for intoxication by CDT. Derl2 is a six-pass transmembrane protein with three predicted loops in the ER lumen [49]. Replacing the third luminal loop from Derl2 with Derl1 sequences supported intoxication, indicating that this loop is not required, though we cannot exclude a more minor role. However, replacing the second luminal loop, which consists of just eight amino acids, two of which are conserved with Derl1, resulted in loss of function. This finding supports a key role for specific amino acids within this small domain in sensitivity to Hd-CDT. The first luminal loop may also be important, though chimeras consisting of this loop from Derl1 swapped with Derl2 and vice versa were not expressed and thus this could not be tested directly. However, replacing the first 88 N-terminal residues, inclusive of the first two transmembrane domains and the first luminal loop, with those from Derl1 did express well but failed to support Hd-CDT intoxication. This N-terminal region also contains the WR motif conserved among rhomboid proteases and required in Derl1 for retrotranslocation of misfolded proteins. However, the WR motif is conserved between Derl1 and Derl2 and point mutations within this WR motif in Derl2 still supported intoxication. These findings suggest that another functional domain exists within this region that is required for intoxication by Hd-CDT. Further studies are needed to determine whether additional requirements for intoxication map to the first luminal loop, the two transmembrane domains, or perhaps the N-terminal tail that extends into the cytosol.

In addition to identifying Derl2, Hrd1, and p97 as host factors usurped by CDTs to exit the ER, the studies presented here provide insight into the mechanism by which Derlin-GFP fusions act as dominant negative proteins. These constructs have been used to study the role of derlin family members in retrograde translocation of misfolded proteins, cytomegalovirus mediated degradation of class I MHC, infection by murine polyomavirus, and intoxication by ricin and cholera toxin [25], [30], [48], [49], [55]; however, the mechanism by which these constructs inhibit ERAD function was unknown. Interestingly, overexpression of Derl1-GFP or Derl2-GFP was previously shown to have no effect on the intoxication of HeLa cells by ricin or Hd-CDT, leading the authors to conclude that derlins are not required for these toxins [22], [25]. Similarly, we found that overexpression of Derl2-GFP (Fig. 4b) or Derl1-GFP (not shown) had no effect on CDT intoxication of parental A745TKR cells. Rather, overexpression of Derl2-GFP actually complemented sensitivity to Hd-CDT in Derl2-deficient CHO-CDT^R^C1 cells. Expression of Derl2ΔC complemented resistance to both ricin and CDT. The data presented here suggest that Derlin-GFP constructs act in a dominant negative manner by blocking interactions mediated by the C-terminus such as SHP box-mediated interactions with p97, and therefore may only exert dominant negative effects on ERAD and trafficking processes that require these interactions. Interestingly, although the interaction of p97 with Derl2 is not required for CDT interaction, p97 activity is indeed required for intoxication as expression of dominant negative p97 causes reduced sensitivity to Hd-CDT. p97 may supply energy for the retrotranslocation process that is common to both misfolded proteins and CDT through interactions with other proteins such as Hrd1 [50], or may be required for other entry or trafficking steps [56]. Determining the precise roles for this multifunctional protein requires more detailed studies and it remains possible that p97 contributes to more than one step in the intoxication pathway.

Previous somatic cell genetic screens identified twelve host genes required for intoxication by CDTs and ricin, but failed to identify Derl2, Hrd1 or p97 [41], [42]. The reason for this difference is unclear, though any single genetic model system is unlikely to provide a complete picture of such a complex biological process. Indeed, the host genes identified thus far only begin to explain the host processes required for cellular binding and entry by CDTs [21], [41], [42]. Only ten of the fifteen host factors identified thus far are required for intoxication by more than one CDT and of these, only two, sphingomyelin synthase 1 (SGSM1) [42] and Derl2 (Fig. 1, 2) have been shown to be required for all four CDTs tested here. These results suggest that various members of the CDT family have evolved distinct strategies to gain access to the host nucleus [21], [57]. Cj-CDT is the most evolutionarily divergent CDT studied here and displays unique requirements for host factors compared with Ec-, Aa-, and Hd-CDTs [42], [57]. Consistent with these prior findings, Cj-CDT had the least dependence on Derl2 and no requirement for Hrd1 (Fig. 2d). Future studies will likely identify many more host requirements for this family of toxins and provide further insight into their cellular entry pathways. Comparison of multiple members of the CDT family will elucidate a core set of host factors required for entry of all CDTs, but will also provide insight into unique solutions evolved by distinct CDTs to gain access to the host nucleus.

## Materials and Methods

### Cell culture

Chinese hamster ovary cells (CHO) and derivatives were maintained in F-12 media (Gibco) supplemented with 10% fetal bovine serum (Sigma Aldrich), 100 U/mL penicillin, 100 µg/mL streptomycin, 5 mM L-glutamine (Invitrogen) and 1 µg/mL doxycycline (Sigma Aldrich). HeLa and 293 cells (American Type Culture Collection) were maintained in Dulbecco's Modified Eagle Medium (DMEM; Cellgro) containing 25 mM HEPES, 4.5 g/L sodium pyruvate, 4.5 g/L glucose, 10% fetal bovine serum (Sigma Aldrich), 100 U/mL penicillin, 100 µg/mL streptomycin, and 5 mM L-glutamine (Invitrogen). In some cases, 293 culture medium was supplemented with 1% non-essential amino acids (Gibco). All cells were cultured at 37°C in a humid atmosphere containing 5% CO_2_.

### Selection of CDT-resistant clones

To isolate chemically mutagenized CDT-resistant clones, ten pools of CHO-pgs A745 cells (A745, provided by Jeff Esko, UCSD) were treated with ICR191 (Sigma Aldrich) at a concentration high enough to kill 90% of the cells [58]. The resulting cells were counted, seeded at 1×10^6^ cells per 10 cm plate and selected with 20 nM Aa-CDT. Resulting resistant cells were subjected to limiting dilutions to obtain single cell clones, expanded and reselected with Aa-CDT.

Selection of retrovirally mutagenized CDT-resistant clones was performed similar to a previously reported protocol [44]. Briefly, an Hd-CDT-sensitive clonal A745 cell line expressing tetR-KRAB (A745TKR) was established. Ten pools of 1×10^6^ A745TKR parental cells were mutagenized by transduction with murine leukemia virus encoding the transcription response element TetO_7_ in the long terminal repeat (pCMMP.GFP-NEO-TRE) at a multiplicity of infection of 0.1. These pools were transcriptionally repressed at proviral integration sites for 96 hours in the absence of doxycycline then selected with 5 nM Hd-CDT for 24 hours. After selection, two of the ten pools yielded colonies; these colonies were picked, expanded and reselected with Hd-CDT. None of the CDT-resistant clones displayed doxycycline dependant sensitivity to CDT, so they were further maintained in the presence of doxycycline.

### Intoxication assays

Mammalian cells were trypsinized, counted and seeded at approximately 1×10^3^ cells per well in 384-well plates. The following day, medium was removed and toxin containing medium was added for 48 hours, followed by addition of ATPlite 1-step reagent (Perkin Elmer). Recombinant CDTs were cloned, expressed, and purified as described previously [57] and ricin was purchased commercially (List Biological Laboratories). Each biological replicate intoxication was performed in triplicate. Analysis of intoxication was performed either by quantitation of pH2AX immunofluorescence (as described previously [57]) or by using ATPlite reagent (Perkin Elmer) according to manufacturer recommendations. Intoxication data obtained by ATPlite reagent was normalized by dividing the luminescence relative light unit (RLU) signal of each replicate by the average of the unintoxicated control cells. All intoxication results presented are representative of at least three biological replicates.

### Sequence capture mediated inverse PCR

In order to identify the location of the provirus in the CDT-resistant clones, genomic DNA was purified from each clone according to manufacturer recommendations (Qiagen), followed by digestion of 2 µg of genomic DNA with BamHI restriction enzyme (New England Biolabs). Digested genomic DNA was purified by column chromatography (Qiagen) and resuspended in 100 mM Tris-HCl, 150 mM NaCl, 50 mM EDTA, pH 7.5, containing 10 pmol biotinylated oligonucleotide complimentary to the 3′ pCMMP long terminal repeat (Sigma Aldrich; [Biotin]GTACCCGTGTTCTCAATAAACCCTC). The samples were heated to 95°C for 5 minutes then plunged on ice, followed by end over end rotation at 55°C for 14 hours.

Streptavidin coated magnetic beads were washed three times with 10 mM Tris-HCl, 2 M NaCl, 1 mM EDTA, pH 7.5 and added to the samples. Samples were vortexed for 0.5 hours at room temperature then the beads were immobilized on a magnet and supernatant removed, followed by three washes with 5 mM Tris-HCl, 1 M NaCl, 0.5 mM EDTA, pH 7.5 and resuspension in 100 µL water. The tubes were heated to 95°C in the presence of the magnet and the supernatant was removed and self-circularized with T4 DNA ligase according to manufacturer recommendations (Fermentas). PCR was performed using the following primers (GAGGGTTTATTGAGAACACGGGTAC and GTGATTGACTACCCGTCAGCGGGGTC) followed by nested PCR with the following primers (CGAGACCACGAGTCGGATGCAACTGC and GTTCCTTGGGAGGGTCTCCTCTG). Amplicons were run in a 1% agarose gel, bands were cut out, column purified (Qiagen) and sequenced (Genewiz).

In order to confirm that the MLV proviral integration occurred at the Derl2 locus, PCR amplification was performed on the genomic DNA from the retrovirally induced CDT resistant clones and the parental A745TKR cells. The primers used for amplification annealed to the fifth exon in the Derl2 open reading frame (CCATGAGCACCCAGGGCAGG) and either forward proviral elements (TGATCGCGCTTCTCGTTGGG) or reverse proviral elements (AGCGCATCGCCTTCTATCGC).

### Subcloning and expression of Derlins

Murine Derl1 and Derl2 cDNA were subcloned by PCR amplifying using the following primers (restriction sites and kozak consensus sequences shown underlined and capitalized, respectively): Derl1 forward aaaagatctTCCACCATGtcggacatcggggactggttcagg; Derl1 reverse aaactcgagctggtctccaagtcggaagc; Derl2 forward aaaagatctTCCACCATGgcgtaccagagcctccggctgg; Derl2 reverse aaactcgagcccaccaaggcgctggccctcacc. The amplicons and the empty retroviral vector pMSCVpuro (Clontech) were digested with BglII and XhoI (New England Biolabs), gel purified (Qiagen) and ligated with T4 DNA ligase (Fermentas). The Gibson assembly reaction was utilized to construct the chimeric Derl1:Derl2 and Derl2:Derl1 [59]. Briefly, primers (Table S1) were designed to span the ends of the segments to be cloned by using the NEBuilder (TM) tool (New England Biolabs). PCR amplification and gel purification were performed to isolate segments to be cloned. Segments were assembled and cloned into pMSCVhygro (Clontech) by using Gibson assembly mastermix according to manufacturer's protocol (New England Biolabs). In order to generate retroviral vectors, plasmid DNA was purified and transfected into human 293 cells along with MLV gag/pol and vesicular stomatitis virus G-spike protein expression plasmids, as previously described [44]. 48 and 72 hours later, resulting retroviral particles were harvested, filter sterilized and used to transduce target cells in the presence of 8 µg/mL polybrene (Sigma Aldrich).

### Immunoprecipitation western blot

Approximately 1×10^7^ cells were lysed in 1% digitonin, 25 mM Tris-HCl, 150 mM NaCl, 5 mM EDTA, 1 U/mL DNAse (Promega), and protease inhibitors (Roche), pH 7.0. The lysates were centrifuged at 14,000× G and supernatants were mixed with either 1 µg/mL rabbit α-Derl2 antibody or 5 µg/mL mouse anti-Hrd1/SYVN1 monoclonal antibody (Sigma Aldrich) and incubated overnight at 4°C with agitation. Protein-A sepharose beads (Santa Cruz Biotechnology) were washed, blocked with 5% bovine serum albumin (EMD Millipore) and incubated with the lysates for 1 hour at room temperature with agitation. Following incubation, the beads were washed three times, mixed with SDS reducing buffer and subjected to SDS-PAGE followed by transfer to PVDF membranes. Membranes were probed with either rabbit anti-Derl2 antibody (Sigma Aldrich) or rabbit anti-Hrd1 polyclonal antibody (Novus Biologicals) at a 1∶2000 dilution followed by HRP conjugated α-rabbit antibody (Invitrogen) to allow detection.

To test interactions between Derl2 and p97, 293 cells were seeded at 1×10^6^ per 10 cm plate and allowed to adhere overnight. The following day, cells were transfected with 10 µg of plasmid DNA by calcium phosphate method. Seventy-two hours post-transfection, the cells were lysed in 1% digitonin lysis buffer (as described above). S-protein agarose beads were blocked in 5% bovine serum albumin for 1 hour and incubated with the lysates overnight at 4°C. The beads were washed with 0.1% digitonin, 25 mM Tris-HCl, 150 mM NaCl, 5 mM EDTA, pH 7.0 and protease inhibitors and then mixed with 1X SDS reducing buffer. Samples were subjected to SDS-PAGE, transferred to PVDF membranes then probed with rabbit anti-S-tag antibody (Cell Signal Technologies) and mouse anti-p97 antibody (Santa Cruz Biotechnology).

### CRISPR mediated knockout of Derl2 and Hrd1

One hundred thousand Hela or 293 cells were transfected with 1 µg Cas9 expression plasmid (AddGene) [45] and 1 µg DNA derived from RT-PCR amplification of gRNA (Integrated DNA Technologies; Derl2 target sequence: AAGAAGTTCATGCGGACAT; Hrd1 target sequence: TGATGGGCAAGGTGTTCTT) using lipofectamine 2000 (Invitrogen) according to the manufacturer's protocol in a 12-well plate. Twenty four hours following transfection, cell culture medium was aspirated and replaced with complete DMEM containing 300 µg/mL of G418 to select for cells successfully transfected with the human codon optimized pcDNA3.3 TOPO vector carrying the Cas9 gene sequence and neomycin resistance cassette. After 72–96 hours under G418 selection the remaining viable cells were expanded to 10 cm tissue culture plates in complete DMEM without G418 and allowed to reach ∼80% confluence, after which toxin resistant cells were selected by intoxication with 5 nM Hd-CDT holotoxin. Cells surviving Hd-CDT intoxication were further expanded and the loss of either Derl2 or Hrd1 was confirmed by IP-western blot.

### Fluorescence microscopy

8 well-chambered slides (Nunc) were seeded with cells and allowed to adhere overnight. The following day, they were chilled on ice for 30 minutes then incubated on ice with 100–200 nM Hd-CDT for 30 minutes. The monolayers were washed with ice-cold PBS pH 7.4 (Lonza), and then incubated at 37°C with complete medium. After 60 minutes at 37°C, the cells were washed with ice-cold PBS pH 7.4, and fixed with ice-cold 2% formaldehyde (Sigma). After fixing for 30 minutes at room temperature, the cells were permeabilized by incubating in PBS 7.4 containing 0.1% Triton X-100 for 15 min, and blocked with 3% BSA (Sigma) for 30 minutes. To probe for Hd-CdtB, cells were incubated with rabbit polyclonal anti-Hd-CdtB antibodies (generated by The Immunological Resource Center, University of Illinois, Urbana, IL) at 4°C overnight, followed by incubation with goat anti-rabbit antibody labeled with either Alexa Fluor 488 or Alexa Fluor 568 (Invitrogen) at room temperature for 2 hours. Where indicated, the ER is labeled with either Alexa Fluor 594 conjugated Concanavalin A (Invitrogen) or mouse monoclonal anti-calreticulin antibody (Abcam) at 4°C overnight, followed by incubation with goat anti-mouse Alexa Fluor 647-labeled antibody (Invitrogen). Where indicated, nuclear counterstaining was performed by either incubating with DAPI for 30 minutes at room temperature or transfecting with 1 µg of plasmid encoding Histone-GFP (pH2B-GFP; Addgene, Cambridge, MA). The slides were mounted with ProLong Gold antifade reagent (Invitrogen) and images were collected using DIC/fluorescence microscopy and deconvoluted by using SoftWoRX constrained iterative deconvolution tool (ratio mode), and analyzed using Imaris 5.7 (Bitplane AG). For each cell, images were collected from an average of 30 z-planes, each at a thickness of 0.2 µm. Nuclear localization analysis was conducted by using the DeltaVision SoftWoRx 3.5.1 software suite. For nuclear localization, the percentage of Hd-CdtB localization into nucleus in parental and Derl2 deficient cells were calculated from approximately 30 cells from each group over at least two independent experiments. To test the colocalization of Hd-CdtB with the endoplasmic reticulum, results were expressed as the localization index, which was derived from calculating the Pearson's coefficient of correlation values, which represent the colocalization of Hd-CdtB and the ER in each z plane of the cell. In these studies, a localization index value of 1.0 indicates 100% localization of Hd-CdtB to the ER, whereas a localization index of 0.0 indicates the absence of Hd-CdtB localization to the ER. The localization index was calculated from the analysis of a total of 30 images collected over at least two independent experiments.

### Dominant negative p97 expression

One hundred thousand 293 cells expressing T-cell receptor alpha fused to green fluorescent protein were seeded the day prior to transfection with 1 µg of plasmid encoding either dominant negative p97 (R586A) or control p97 (R700A) co-expressed with CD4 as a surface marker of positive expression (plasmids generously provided by Ron Kopito, Stanford University). Seventy-two hours after transfection, the cells were intoxicated with a concentration of Hd-CDT sufficient to cause cell cycle arrest in 48 hours. Intoxicated cells were rinsed with PBS, detached from the wells with PBS+1 mM EDTA, rinsed with PBS again and incubated with phycoerythrin conjugated rabbit anti-CD4 antibody (Invitrogen) in PBS+3% bovine serum albumin on ice for 30 minutes. Following staining, the cells were washed with PBS, fixed with 1% formaldehyde, washed with PBS again and stained with Hoechst 33342 for 10 minutes. Cells were then washed with PBS, resuspended in PBS and analyzed for phycoerythrin, Hoechst and GFP fluorescence by flow cytometry (LSR II; Becton Dickinson). Cell cycle analysis was performed on CD4 expressing cells.

### Statistical analysis

The half maximal lethal dose (LD_50_) of ricin intoxication was calculated by log transforming ricin concentrations and calculating sigmoidal variable slope dose response curves using the least squares (ordinary) fitting method. Paired t-tests were performed on average LD_50_ values calculated from three independent experiments performed in triplicate to determine two tailed p-values. Data analysis was performed using Prism version 5.0d (GraphPad software).

## Supporting Information

Figure S1 **CHO-CDT^R^C1 and CHO-CDT^R^F1 cells display reduced Hd-CDT-mediated cell cycle arrest.** Parental A745TKR and Derl2 deficient CHO-CDT^R^C1 and CHO-CDT^R^F1 cells were intoxicated with Hd-CDT for 48 hours, stained with propidium iodide and analyzed by flow cytometry for cell cycle. Data graphed is percent of the cell population in G2.
(TIFF)

Figure S2 **CHO-CDT^R^F1 cell line is resistant to CDT.** Viability of parental A745TKR cells, retrovirally induced mutant CHO-CDT^R^F1 cells, and CHO-CDT^R^F1 cells expressing Derl2 after intoxication with Aa-CDT (a), Hd-CDT (b), Ec-CDT (c) and Cj-CDT (d). Intoxication was performed similar to figure 1, data are representative of at least three independent experiments performed in triplicate, percent viability is normalized to unintoxicated controls and error bars indicate standard error.
(TIFF)

Figure S3 **CDT trafficking in the CHO-CDT^R^F1 cell line is blocked at the ER.** (a) CHO-CDT^R^F1 cells were incubated with Hd-CDT on ice, washed and incubated at 37°C for 10 or 60 minutes. Cells were then fixed and stained with DAPI (nuclei, blue), Concanavalin A (ER, red) and anti-Hd-CdtB (green) antibody. White scale bars indicate 5 µm. (b,c) Quantification of microscopy results comparing the percentage of cells with at least one green puncta localized to the nucleus or Pearson's coefficient values indicating colocalization of the Hd-CdtB signal with the ER marker. Images and quantitation are representative of those collected from a total of 30 randomly chosen cells analyzed during three independent experiments and error bars represent standard deviations. Data for parental A745TKR cells from figure 3 is reproduced here for comparison.
(TIFF)

Figure S4 **ΔHrd1 cells display reduced Hd-CDT-mediated cell cycle arrest.** Wildtype 293 and 293 ΔHrd1 cells were intoxicated with Hd-CDT for 48 hours, stained with propidium iodide and analyzed by flow cytometry for cell cycle distribution. Data from three independent experiments is graphed as percent of the cell population in G2.
(TIFF)

## References

[1] Ahmed HJ, Svensson LA, Cope LD, Latimer JL, Hansen EJ, et al (2001) Prevalence of cdtABC genes encoding cytolethal distending toxin among Haemophilus ducreyi and Actinobacillus actinomycetemcomitans strains. J Med Microbiol 50: 860–864. 11599734 10.1099/0022-1317-50-10-860

[2] McAuley JL, Linden SK, Png CW, King RM, Pennington HL, et al (2007) MUC1 cell surface mucin is a critical element of the mucosal barrier to infection. J Clin Invest 117: 2313–2324. 17641781 10.1172/JCI26705 PMC1913485

[3] Young VB, Knox KA, Pratt JS, Cortez JS, Mansfield LS, et al (2004) In vitro and in vivo characterization of Helicobacter hepaticus cytolethal distending toxin mutants. Infect Immun 72: 2521–2527. 15102759 10.1128/IAI.72.5.2521-2527.2004 PMC387909

[4] Fox JG, Rogers AB, Whary MT, Ge Z, Taylor NS, et al (2004) Gastroenteritis in NF-kappaB-deficient mice is produced with wild-type Camplyobacter jejuni but not with C. jejuni lacking cytolethal distending toxin despite persistent colonization with both strains. Infect Immun 72: 1116–1125. 14742559 10.1128/IAI.72.2.1116-1125.2004 PMC321575

[5] Purdy D, Buswell CM, Hodgson AE, McAlpine K, Henderson I, et al (2000) Characterisation of cytolethal distending toxin (CDT) mutants of Campylobacter jejuni. J Med Microbiol 49: 473–479. 10798561 10.1099/0022-1317-49-5-473

[6] Ge Z, Rogers AB, Feng Y, Lee A, Xu S, et al (2007) Bacterial cytolethal distending toxin promotes the development of dysplasia in a model of microbially induced hepatocarcinogenesis. Cell Microbiol 9: 2070–2080. 17441986 10.1111/j.1462-5822.2007.00939.x

[7] Ge Z, Feng Y, Whary MT, Nambiar PR, Xu S, et al (2005) Cytolethal distending toxin is essential for Helicobacter hepaticus colonization in outbred Swiss Webster mice. Infect Immun 73: 3559–3567. 15908385 10.1128/IAI.73.6.3559-3567.2005 PMC1111878

[8] Gargi A, Reno M, Blanke SR (2012) Bacterial toxin modulation of the eukaryotic cell cycle: are all cytolethal distending toxins created equally? Front Cell Infect Microbiol 2: 124. 23061054 10.3389/fcimb.2012.00124 PMC3465861

[9] Shenker BJ, Hoffmaster RH, Zekavat A, Yamaguchi N, Lally ET, et al (2001) Induction of apoptosis in human T cells by Actinobacillus actinomycetemcomitans cytolethal distending toxin is a consequence of G2 arrest of the cell cycle. J Immunol 167: 435–441. 11418680 10.4049/jimmunol.167.1.435

[10] Pickett CL, Whitehouse CA (1999) The cytolethal distending toxin family. Trends Microbiol 7: 292–297. 10390639 10.1016/s0966-842x(99)01537-1

[11] Guidi R, Guerra L, Levi L, Stenerlow B, Fox JG, et al (2013) Chronic exposure to the cytolethal distending toxins of Gram-negative bacteria promotes genomic instability and altered DNA damage response. Cell Microbiol 15: 98–113. 22998585 10.1111/cmi.12034 PMC4136655

[12] Guerra L, Cortes-Bratti X, Guidi R, Frisan T (2011) The biology of the cytolethal distending toxins. Toxins (Basel) 3: 172–190. 22069704 10.3390/toxins3030172 PMC3202825

[13] Thelestam M, Frisan T (2004) Cytolethal distending toxins. Rev Physiol Biochem Pharmacol 152: 111–133. 15338430 10.1007/s10254-004-0030-8

[14] Blanke SR (2006) Portals and Pathways: Principles of Bacterial Toxin Entry into Host Cells. Microbe 1: 26–32.

[15] Elwell CA, Dreyfus LA (2000) DNase I homologous residues in CdtB are critical for cytolethal distending toxin-mediated cell cycle arrest. Mol Microbiol 37: 952–963. 10972814 10.1046/j.1365-2958.2000.02070.x

[16] Lara-Tejero M, Galan JE (2000) A bacterial toxin that controls cell cycle progression as a deoxyribonuclease I-like protein. Science 290: 354–357. 11030657 10.1126/science.290.5490.354

[17] McSweeney LA, Dreyfus LA (2005) Carbohydrate-binding specificity of the Escherichia coli cytolethal distending toxin CdtA-II and CdtC-II subunits. Infect Immun 73: 2051–2060. 15784546 10.1128/IAI.73.4.2051-2060.2005 PMC1087409

[18] Cao L, Bandelac G, Volgina A, Korostoff J, DiRienzo JM (2008) Role of aromatic amino acids in receptor binding activity and subunit assembly of the cytolethal distending toxin of Aggregatibacter actinomycetemcomitans. Infect Immun 76: 2812–2821. 18426882 10.1128/IAI.00126-08 PMC2446717

[19] Cao L, Volgina A, Huang CM, Korostoff J, DiRienzo JM (2005) Characterization of point mutations in the cdtA gene of the cytolethal distending toxin of Actinobacillus actinomycetemcomitans. Mol Microbiol 58: 1303–1321. 16313618 10.1111/j.1365-2958.2005.04905.x PMC1435350

[20] Nesic D, Stebbins CE (2005) Mechanisms of assembly and cellular interactions for the bacterial genotoxin CDT. PLoS Pathog 1: e28. 16304609 10.1371/journal.ppat.0010028 PMC1287909

[21] Gargi A, Tamilselvam B, Powers B, Prouty MG, Lincecum T, et al (2013) Cellular interactions of the cytolethal distending toxins from escherichia coli and haemophilus ducreyi. J Biol Chem 288 11: 7492–505. 23306199 10.1074/jbc.M112.448118 PMC3597790

[22] Guerra L, Teter K, Lilley BN, Stenerlow B, Holmes RK, et al (2005) Cellular internalization of cytolethal distending toxin: a new end to a known pathway. Cell Microbiol 7: 921–934. 15953025 10.1111/j.1462-5822.2005.00520.x

[23] Spooner RA, Watson PD, Marsden CJ, Smith DC, Moore KA, et al (2004) Protein disulphide-isomerase reduces ricin to its A and B chains in the endoplasmic reticulum. Biochem J 383: 285–293. 15225124 10.1042/BJ20040742 PMC1134069

[24] Day PJ, Owens SR, Wesche J, Olsnes S, Roberts LM, et al (2001) An interaction between ricin and calreticulin that may have implications for toxin trafficking. J Biol Chem 276: 7202–7208. 11113144 10.1074/jbc.M009499200

[25] Slominska-Wojewodzka M, Gregers TF, Walchli S, Sandvig K (2006) EDEM is involved in retrotranslocation of ricin from the endoplasmic reticulum to the cytosol. Mol Biol Cell 17: 1664–1675. 16452630 10.1091/mbc.E05-10-0961 PMC1415288

[26] Moreau D, Kumar P, Wang SC, Chaumet A, Chew SY, et al (2011) Genome-wide RNAi screens identify genes required for Ricin and PE intoxications. Dev Cell 21: 231–244. 21782526 10.1016/j.devcel.2011.06.014

[27] Li S, Spooner RA, Allen SC, Guise CP, Ladds G, et al (2010) Folding-competent and folding-defective forms of ricin A chain have different fates after retrotranslocation from the endoplasmic reticulum. Mol Biol Cell 21: 2543–2554. 20519439 10.1091/mbc.E09-08-0743 PMC2912342

[28] Redmann V, Oresic K, Tortorella LL, Cook JP, Lord M, et al (2011) Dislocation of ricin toxin A chains in human cells utilizes selective cellular factors. J Biol Chem 286: 21231–21238. 21527639 10.1074/jbc.M111.234708 PMC3122183

[29] Simpson JC, Roberts LM, Romisch K, Davey J, Wolf DH, et al (1999) Ricin A chain utilises the endoplasmic reticulum-associated protein degradation pathway to enter the cytosol of yeast. FEBS Lett 459: 80–84. 10508921 10.1016/s0014-5793(99)01222-3

[30] Bernardi KM, Forster ML, Lencer WI, Tsai B (2008) Derlin-1 facilitates the retro-translocation of cholera toxin. Mol Biol Cell 19: 877–884. 18094046 10.1091/mbc.E07-08-0755 PMC2262961

[31] Dixit G, Mikoryak C, Hayslett T, Bhat A, Draper RK (2008) Cholera toxin up-regulates endoplasmic reticulum proteins that correlate with sensitivity to the toxin. Exp Biol Med (Maywood) 233: 163–175. 18222971 10.3181/0705-RM-132

[32] Hebert DN, Bernasconi R, Molinari M (2010) ERAD substrates: which way out? Semin Cell Dev Biol 21: 526–532. 20026414 10.1016/j.semcdb.2009.12.007

[33] Jarosch E, Taxis C, Volkwein C, Bordallo J, Finley D, et al (2002) Protein dislocation from the ER requires polyubiquitination and the AAA-ATPase Cdc48. Nat Cell Biol 4: 134–139. 11813000 10.1038/ncb746

[34] Rabinovich E, Kerem A, Frohlich KU, Diamant N, Bar-Nun S (2002) AAA-ATPase p97/Cdc48p, a cytosolic chaperone required for endoplasmic reticulum-associated protein degradation. Mol Cell Biol 22: 626–634. 11756557 10.1128/MCB.22.2.626-634.2002 PMC139744

[35] Ye Y, Shibata Y, Yun C, Ron D, Rapoport TA (2004) A membrane protein complex mediates retro-translocation from the ER lumen into the cytosol. Nature 429: 841–847. 15215856 10.1038/nature02656

[36] Teter K, Holmes RK (2002) Inhibition of endoplasmic reticulum-associated degradation in CHO cells resistant to cholera toxin, Pseudomonas aeruginosa exotoxin A, and ricin. Infect Immun 70: 6172–6179. 12379695 10.1128/IAI.70.11.6172-6179.2002 PMC130429

[37] Guerra L, Nemec KN, Massey S, Tatulian SA, Thelestam M, et al (2009) A novel mode of translocation for cytolethal distending toxin. Biochim Biophys Acta 1793: 489–495. 19118582 10.1016/j.bbamcr.2008.11.017 PMC2647582

[38] Damek-Poprawa M, Jang JY, Volgina A, Korostoff J, DiRienzo JM (2012) Localization of Aggregatibacter actinomycetemcomitans cytolethal distending toxin subunits during intoxication of live cells. Infect Immun 80: 2761–2770. 22645284 10.1128/IAI.00385-12 PMC3434584

[39] McSweeney LA, Dreyfus LA (2004) Nuclear localization of the Escherichia coli cytolethal distending toxin CdtB subunit. Cell Microbiol 6: 447–458. 15056215 10.1111/j.1462-5822.2004.00373.x

[40] Nishikubo S, Ohara M, Ueno Y, Ikura M, Kurihara H, et al (2003) An N-terminal segment of the active component of the bacterial genotoxin cytolethal distending toxin B (CDTB) directs CDTB into the nucleus. J Biol Chem 278: 50671–50681. 12947116 10.1074/jbc.M305062200

[41] Carette JE, Guimaraes CP, Varadarajan M, Park AS, Wuethrich I, et al (2009) Haploid genetic screens in human cells identify host factors used by pathogens. Science 326: 1231–1235. 19965467 10.1126/science.1178955

[42] Carette JE, Guimaraes CP, Wuethrich I, Blomen VA, Varadarajan M, et al (2011) Global gene disruption in human cells to assign genes to phenotypes by deep sequencing. Nat Biotechnol 29: 542–546. 21623355 10.1038/nbt.1857 PMC3111863

[43] Strausberg RL, Feingold EA, Klausner RD, Collins FS (1999) The mammalian gene collection. Science 286: 455–457. 10521335 10.1126/science.286.5439.455

[44] Banks DJ, Bradley KA (2007) SILENCE: a new forward genetic technology. Nat Methods 4: 51–53. 17179935 10.1038/nmeth991

[45] Mali P, Yang L, Esvelt KM, Aach J, Guell M, et al (2013) RNA-guided human genome engineering via Cas9. Science 339: 823–826. 23287722 10.1126/science.1232033 PMC3712628

[46] Dang H, Klokk TI, Schaheen B, McLaughlin BM, Thomas AJ, et al (2011) Derlin-dependent retrograde transport from endosomes to the Golgi apparatus. Traffic 12: 1417–1431. 21722281 10.1111/j.1600-0854.2011.01243.x PMC4596261

[47] Huang CH, Hsiao HT, Chu YR, Ye Y, Chen X (2013) Derlin2 facilitates HRD1-mediated retro-translocation of sonic hedgehog at the endoplasmic reticulum. J Biol Chem 288 35: 25330–9. 23867461 10.1074/jbc.M113.455212 PMC3757197

[48] Lilley BN, Ploegh HL (2004) A membrane protein required for dislocation of misfolded proteins from the ER. Nature 429: 834–840. 15215855 10.1038/nature02592

[49] Greenblatt EJ, Olzmann JA, Kopito RR (2011) Derlin-1 is a rhomboid pseudoprotease required for the dislocation of mutant alpha-1 antitrypsin from the endoplasmic reticulum. Nat Struct Mol Biol 18: 1147–1152. 21909096 10.1038/nsmb.2111 PMC3196324

[50] Ye Y, Meyer HH, Rapoport TA (2001) The AAA ATPase Cdc48/p97 and its partners transport proteins from the ER into the cytosol. Nature 414: 652–656. 11740563 10.1038/414652a

[51] DeLaBarre B, Christianson JC, Kopito RR, Brunger AT (2006) Central pore residues mediate the p97/VCP activity required for ERAD. Mol Cell 22: 451–462. 16713576 10.1016/j.molcel.2006.03.036

[52] Wang Y, Zhang Y, Ha Y (2006) Crystal structure of a rhomboid family intramembrane protease. Nature 444: 179–180. 17051161 10.1038/nature05255

[53] Wu Z, Yan N, Feng L, Oberstein A, Yan H, et al (2006) Structural analysis of a rhomboid family intramembrane protease reveals a gating mechanism for substrate entry. Nat Struct Mol Biol 13: 1084–1091. 17099694 10.1038/nsmb1179

[54] Sandvig K, van Deurs B (2005) Delivery into cells: lessons learned from plant and bacterial toxins. Gene Ther 12: 865–872. 15815697 10.1038/sj.gt.3302525

[55] Lilley BN, Gilbert JM, Ploegh HL, Benjamin TL (2006) Murine polyomavirus requires the endoplasmic reticulum protein Derlin-2 to initiate infection. J Virol 80: 8739–8744. 16912321 10.1128/JVI.00791-06 PMC1563856

[56] Meyer H, Bug M, Bremer S (2012) Emerging functions of the VCP/p97 AAA-ATPase in the ubiquitin system. Nat Cell Biol 14: 117–123. 22298039 10.1038/ncb2407

[57] Eshraghi A, Maldonado-Arocho FJ, Gargi A, Cardwell MM, Prouty MG, et al (2010) Cytolethal distending toxin family members are differentially affected by alterations in host glycans and membrane cholesterol. J Biol Chem 285: 18199–18207. 20385557 10.1074/jbc.M110.112912 PMC2881744

[58] Bradley KA, Mogridge J, Mourez M, Collier RJ, Young JA (2001) Identification of the cellular receptor for anthrax toxin. Nature 414: 225–229. 11700562 10.1038/n35101999

[59] Gibson DG, Young L, Chuang RY, Venter JC, Hutchison CA 3rd, et al (2009) Enzymatic assembly of DNA molecules up to several hundred kilobases. Nat Methods 6: 343–345. 19363495 10.1038/nmeth.1318

