# Supplementary figures and images for: Cytolethal Distending Toxins Require Components of the ER-Associated Degradation Pathway for Host Cell Entry
Source: PLoS Pathog. 2014 Jul 31;10(7):e1004295. doi: 10.1371/journal.ppat.1004295 (PMC4117610; doi:10.1371/journal.ppat.1004295)

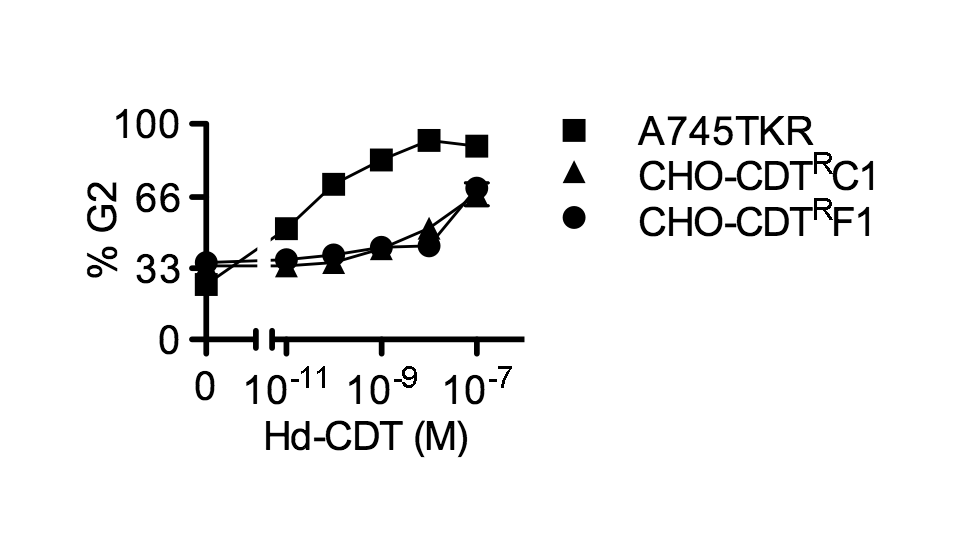

Supplement: Figure S1 — CHO-CDTRC1 and CHO-CDTRF1 cells display reduced Hd-CDT-mediated cell cycle arrest. Parental A745TKR and Derl2 deficient CHO-CDTRC1 and CHO-CDTRF1 cells were intoxicated with Hd-CDT for 48 hours, stained with propidium iodide and analyzed by flow cytometry for cell cycle. Data graphed is percent of the cell population in G2. (TIFF) [file ppat.1004295.s001.tiff]

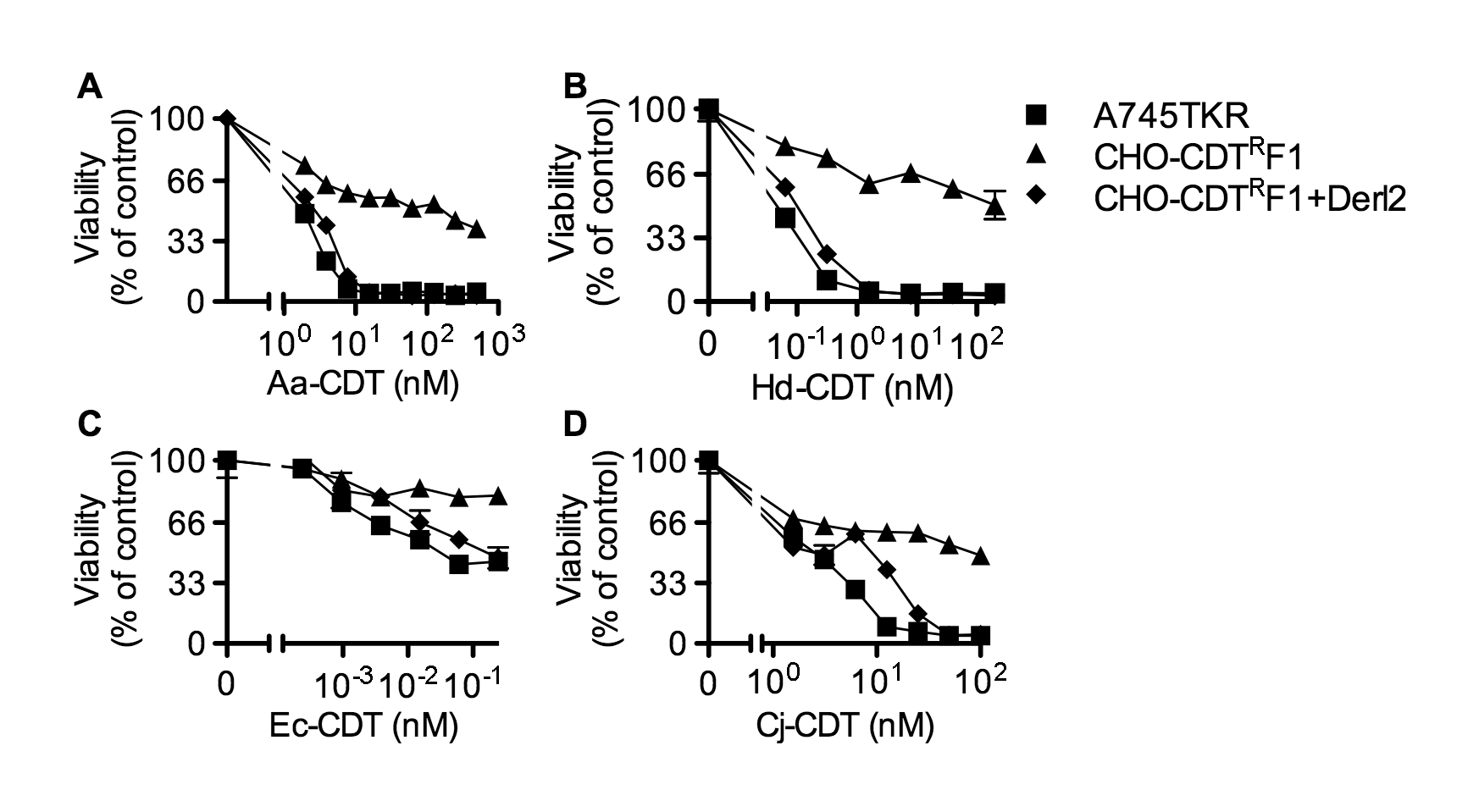

Supplement: Figure S2 — CHO-CDTRF1 cell line is resistant to CDT. Viability of parental A745TKR cells, retrovirally induced mutant CHO-CDTRF1 cells, and CHO-CDTRF1 cells expressing Derl2 after intoxication with Aa-CDT (a), Hd-CDT (b), Ec-CDT (c) and Cj-CDT (d). Intoxication was performed similar to figure 1, data are representative of at least three independent experiments performed in triplicate, percent viability is normalized to unintoxicated controls and error bars indicate standard error. (TIFF) [file ppat.1004295.s002.tiff]

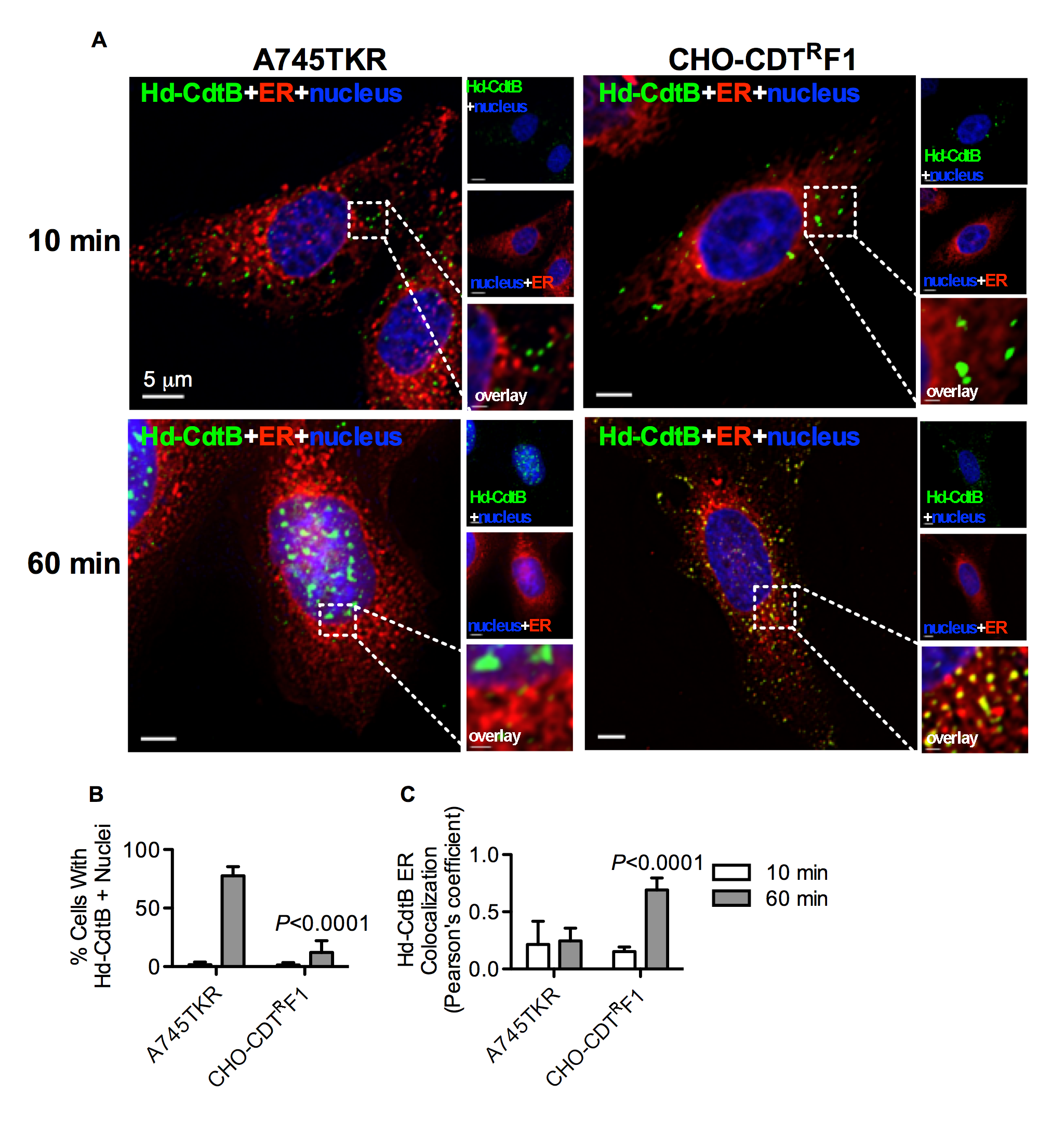

Supplement: Figure S3 — CDT trafficking in the CHO-CDTRF1 cell line is blocked at the ER. (a) CHO-CDTRF1 cells were incubated with Hd-CDT on ice, washed and incubated at 37°C for 10 or 60 minutes. Cells were then fixed and stained with DAPI (nuclei, blue), Concanavalin A (ER, red) and anti-Hd-CdtB (green) antibody. White scale bars indicate 5 µm. (b,c) Quantification of microscopy results comparing the percentage of cells with at least one green puncta localized to the nucleus or Pearson's coefficient values indicating colocalization of the Hd-CdtB signal with the ER marker. Images and quantitation are representative of those collected from a total of 30 randomly chosen cells analyzed during three independent experiments and error bars represent standard deviations. Data for parental A745TKR cells from figure 3 is reproduced here for comparison. (TIFF) [file ppat.1004295.s003.tiff]

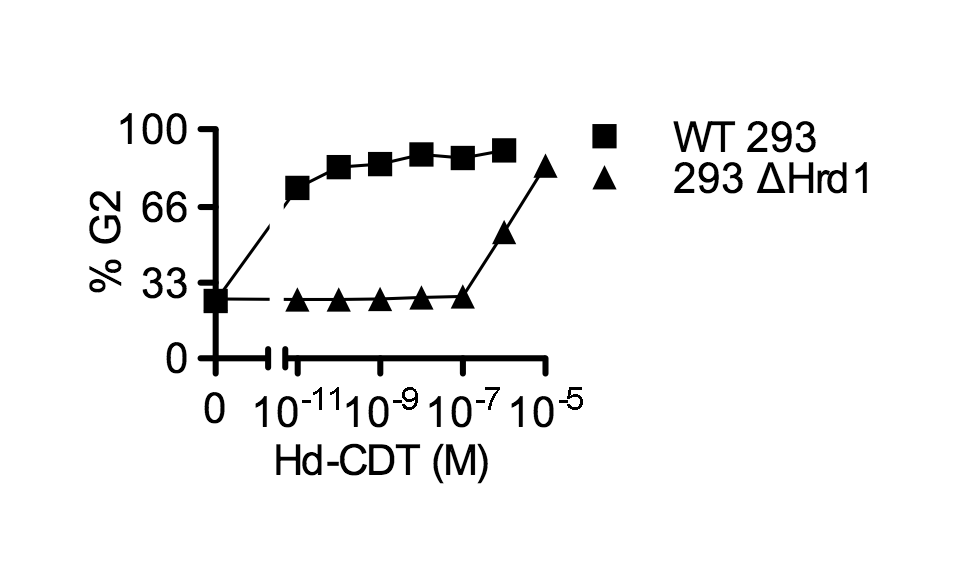

Supplement: Figure S4 — ΔHrd1 cells display reduced Hd-CDT-mediated cell cycle arrest. Wildtype 293 and 293 ΔHrd1 cells were intoxicated with Hd-CDT for 48 hours, stained with propidium iodide and analyzed by flow cytometry for cell cycle distribution. Data from three independent experiments is graphed as percent of the cell population in G2. (TIFF) [file ppat.1004295.s004.tiff]
